# The effects of shifting tasks from pharmacy to non-pharmacy personnel for providing antiretroviral therapy to people living with HIV: a systematic review protocol

**DOI:** 10.1136/bmjopen-2015-008195

**Published:** 2016-03-11

**Authors:** Nyanyiwe M Mbeye, Tamara Kredo, Charles S Wiysonge

**Affiliations:** 1 Cochrane South Africa, South African Medical Research Council, Tygerberg, South Africa; 2 Faculty of Medicine and Health Sciences, Centre for Evidence-based Health Care, Stellenbosch University, Cape Town, South Africa

**Keywords:** antiretroviral therapy, dispensing and distribution, lay providers, pharmacy personnel, task shiftng

## Abstract

**Introduction:**

Shifting selected antiretroviral therapy (ART) tasks from specialised healthcare workers to those with shorter or less formal training has been implemented in resource-limited settings to alleviate critical shortages of human resources for health. However, the specifics of shifting ART dispensing from pharmacy to non-pharmacy personnel have not been addressed in a systematic review, although this can potentially increase access to ART. We will assess the effects of shifting dispensing and distribution of ART and adherence assessment from pharmacy to non-pharmacy personnel in low and middle-income countries.

**Methods and analysis:**

We will search PubMed, CENTRAL, EMBASE, WHO Global Health Library and relevant grey literature for eligible controlled trials. Two authors will screen the search output, select eligible studies, assess risk of bias and extract data from included studies, resolving discrepancies by discussion and consensus. We will perform meta-analysis using both fixed and random effects models, investigate clinical and statistical heterogeneity, and assess our confidence in the overall evidence using standard Cochrane methods, including GRADE.

**Ethics and dissemination:**

Only secondary data will be included in this review and ethics approval is not required. We will disseminate the review findings in various scientific fora, including peer-reviewed journals. The findings may help to inform policy makers in defining the scope of work of healthcare workers, and global recommendations for shifting the dispensing and distribution of ART from pharmacy to non-pharmacy personnel.

**Trial registration number:**

CRD42015017034.

**Strengths and limitations of this study**

- To our knowledge, this is the first published protocol of a systematic review that will investigate the effects of task shifting from pharmacy to non-pharmacy personnel for dispensing or distributing antiretroviral therapy to patients living with HIV.
- The protocol was written according to the PRISMA-P (Preferred Reporting Items for Systematic Review and Meta-Analysis Protocols) recommendations.
- The review findings may help to inform antiretroviral therapy guidelines by the WHO.
- The possible weakness of the planned review would be the limitations of included studies, for example, high risk of bias and heterogeneity of settings, designs and effects.

## Introduction

### Description of the condition

By March 2015, 15 million (40.7%) of the estimated 36.9 million people living with HIV (PLHIV) globally were receiving antiretroviral therapy (ART).^1^ Combination ART is effective for reducing HIV related morbidity and mortality as well as preventing HIV transmission.^2^ Initiating ART early in the course of HIV infection has been associated with better health outcomes, both at patient and population levels.^3^ ^4^ Scale-up of ART in low and middle income countries has averted more than 5 million deaths; however, bottlenecks preventing universal access to ART still exist. One challenge is the critical shortage of human resources for health (HRH), including for the delivery of essential HIV related pharmacy services.

The WHO recommends a minimum of one pharmacist per 2300 population, but most countries in low-resource settings such as sub-Saharan Africa have not yet met this target.^5^ In addition to the absolute shortage, it is likely that there is an uneven distribution of pharmacists in such settings, as is the case with other specialist healthcare workers who tend to concentrate in urban areas and the private sector, further aggravating the HRH shortage.^6^ For instance in South Africa, which is home to the largest number of PLHIV in any country in the world, in 2010 only 24% of registered pharmacists worked in the public sector where 80% of the population received care.^7^

### Description of the intervention

Studies and programme reports indicate that involvement of pharmacy personnel in HIV care results in improved patient outcomes. For instance, in the USA, the use of a multidisciplinary team approach with pharmacists assuming a central role in ART initiation, dispensing and adherence counselling improved treatment outcomes such as viral load, patient retention and medication adherence.^8^

The work of pharmacists includes supply management, dispensing and distributing medications, promoting adherence, identifying and preventing potential medication-related issues, and monitoring and reporting adverse events. In some settings, programmes have implemented alternative models of pharmacy services that shift selected tasks from pharmacy to non-pharmacy personnel. Such alternative models could potentially increase the number of health workers involved in ART distribution, adherence counselling and patient education, free more time for pharmacy personnel, support the integration of ART in primary care settings, minimise the number of facility visits for ART collection, and reduce pharmacy queue waiting times for patients.^9^

However, the specifics of shifting ART related tasks from pharmacy to non-pharmacy personnel have not yet been addressed in a systematic review. We therefore plan to synthesise the evidence for task shifting in pharmacy services, where non-pharmacy personnel undertake ART dispensing and distribution and medication adherence counselling. For this systematic review, pharmacy personnel will include both pharmacists and pharmacy technicians. Pharmacy technicians constitute an important part of the pharmacy workforce in low and middle-income countries: a survey of 26 low and middle-income countries in 2011 revealed that pharmacy technicians constitute 10% (Nigeria) to 70% (Pakistan) of the pharmacy workforce.^10^

### How the intervention might work

Within the last decade, several high HIV burden countries adopted task shifting strategies where nurses and non-physician clinicians initiate and maintain ART.^11^ Although this has undeniably expanded access to ART, it is also increasingly essential that long facility waiting times and frequent facility visits to collect ART are addressed to alleviate the burden of care, both for patients and healthcare providers.^12^ ^13^

Recent studies in Uganda, Kenya and Mozambique have shown positive outcomes when non-health professionals (lay people) delivered ART at the community level.^14^ In Mozambique the use of PLHIV for distributing ART, monitoring adherence, reporting outcomes and referring sick patients to health facilities yielded a retention rate of 97.5% among stable patients on ART.^14^ In a cluster randomised trial in Uganda, the use of trained community health workers produced comparable results with facility-based ART programme in terms of patient retention, viral load suppression and mortality rate.^15^ Similar findings were also obtained in Kenya and Uganda when lay providers were engaged in ART delivery.^15^ ^16^

Task shifting has therefore been seen as an achievable solution to the critical human resource shortages for scale-up of ART.^17^ While it is imperative to increase the rate of recruitment and training of health workers as well as improve working conditions to reduce attrition and emigration, the HIV pandemic requires a more urgent measure to address the critical skills shortage.^18^ Such measures may include shifting selected tasks (including dispensing and distributing ART and adherence counselling) from pharmacy to non-pharmacy personnel. The task shifting could free time for pharmacy personnel to focus on more technical functions such as supply management and pharmacovigilance.

### Why this review is important

Previous systematic reviews of task shifting for increasing ART access focused on clinical services where nurses and non-clinician physicians provide care.^11^ Dependence on and shortages of pharmacists are also key constraints on ART expansion, but the specifics of task shifting for ART dispensing or distribution from pharmacy to non-pharmacy personnel have not been reviewed systematically. We will systematically review the scientific literature and assess the efficacy and safety of task shifting models that use non-pharmacy personnel in dispensing or distributing ART and assessing adherence to treatment of HIV infection.

## Objective

The aim of this review is to evaluate the efficacy and safety of shifting dispensing and distribution of ART as well as assessment of adherence from pharmacy to non-pharmacy personnel.

## Methods

This review protocol has been registered in the PROSPERO International Prospective Register of Systematic Reviews (http://www.crd.york.ac.uk/PROSPERO), registration number CRD42015017034.

### Criteria for considering studies for this review

#### Types of studies

We will include randomised controlled trials (RCTs) and non-RCTs, irrespective of whether allocation to interventions occurred at the individual or cluster level.

#### Types of participants

Participants will be PLHIV receiving ART.

#### Types of interventions

We will include studies that evaluate the shifting of selected tasks from pharmacy personnel to non-pharmacy personnel. The selected tasks include dispensing and distribution of ART and adherence assessment. Pharmacy personnel will include both pharmacists and pharmacy technicians. Non-pharmacy personnel may include (but are not limited to) nurses, non-physician clinicians, and lay providers such as patient peer groups, community volunteers, PLHIV and community health committees.

#### Types of outcome measures

##### Primary outcomes

The primary outcome for this review is risk of death.

##### Secondary outcomes

Our secondary outcome measures include:

- Virological suppression
- Number of all-cause sick visits made to the health facility, including for adverse events
- Loss to follow-up
- Adherence to ART (as measured within the study, eg, pill counts, recall methods, digital methods)
- Acceptability to pharmacy personnel, non-pharmacy personnel and patients
- Harm, including error rates.

### Search methods for identification of studies

We will perform a comprehensive and exhaustive search of electronic databases and conference proceedings in an attempt to identify all relevant studies available by the search date, regardless of language of publication or publication status (published, unpublished, in press or in progress).

### Databases of peer-reviewed literature

We will search the following electronic databases, from 1 January 1996 to the search date:

- Cochrane Central Register of Controlled Trials (CENTRAL)
- Excerpta Medica Database (EMBASE)
- PubMed
- ISI Web of Science (Science Citation index)
- WHO Global Health Library, which includes references from AIM (AFRO), LILACS (AMRO/PAHO), IMEMR (EMRO), IMSEAR (SEARO) and WPRIM (WPRO).

Along with appropriate Medical Subject Heading (MeSH) terms and relevant keywords, we will use the Cochrane Highly Sensitive Search Strategy for identifying reports of randomised controlled trials in MEDLINE,^19^ and the Cochrane validated strategies for identifying references relevant to HIV infection and AIDS. To identify other study designs, the RCT string will be omitted. The search strategy will be iterative in that references of included studies will be searched for additional references. See table 1 for our provisional search strategy for electronic databases.

**Table 1:** Proposed search strategy for electronic databases

ID	Search terms
PubMed	
#1	(task*[tiab] OR task-shifting[tiab] OR referr*[tiab] OR referral and consultation[mh] OR role*[tiab]) AND (health personnel[mh] OR doctor[tiab] OR doctors[tiab] OR clinician[tiab] OR clinicians[tiab] OR physician[tiab] OR physicians[tiab] OR “healthcare provider"[tiab] OR “healthcare providers"[tiab] OR “health care provider"[tiab] OR “health care providers"[tiab] OR pharmac*[tiab] OR apothecar*[tiab] OR chemist*[tiab] OR dispensar*[tiab])
#2	randomized controlled trial[pt] OR controlled clinical trial[pt] OR randomized controlled trials[MeSH] OR random allocation[MeSH] OR double-blind method[MeSH] OR single-blind method[MeSH] OR clinical trial[pt] OR clinical trials[MeSH] OR (“clinical trial"[tw]) OR ((singl*[tw] OR doubl*[tw] OR trebl*[tw] OR tripl*[tw]) AND (mask*[tw] OR blind*[tw])) OR random*[tw] OR research design[mh:noexp] OR prospective studies[MeSH] OR control*[tw] OR volunteer*[tw]) OR observational[tw] OR non-random*[tw] OR nonrandom*[tw] OR before after study[tw] OR time series[tw] OR cohort*[tw] OR cross-section*[tw] OR prospective*[tw] OR retrospective*[tw] OR research design[mh:noexp] OR follow-up studies[MeSH] OR longitud*[tw] OR evaluat*[tiab] OR pre-post[tw] OR (pre-test[tw] AND post-test[tw]) NOT (animals[MeSH] NOT human[MeSH])
#3	(HIV Infections[MeSH] OR HIV[MeSH] OR hiv[tiab] OR hiv-1[tiab] OR hiv-2*[tiab] OR hiv1[tiab] OR hiv2[tiab] OR hiv infect*[tiab] OR human immunodeficiency virus[tiab]OR human immune deficiency virus[tiab] OR human immuno-deficiency virus[tiab] OR human immune-deficiency virus[tiab] OR ((human immun*) AND(deficiency virus[tiab])) OR acquired immunodeficiency syndromes[tiab] OR acquired immune deficiency syndrome[tiab] OR acquired immuno-deficiency syndrome[ tiab] OR acquired immune-deficiency syndrome[ tiab] OR ((acquired immun*) AND (deficiency syndrome[tiab])) or “sexually transmitted diseases, viral”[mh]) OR HIV[tiab] OR HIV/AIDS[tiab] OR HIV-infected[tiab] OR HIV[title] OR HIV/AIDS[title] OR HIV-infected[title]
#4	(HAART[tiab] OR ART[tiab] OR cART[tiab] OR antiretroviral[tiab] OR anti-retroviral[tiab] OR anti-viral[tiab] OR antiviral[tiab] OR “Antiretroviral Therapy, Highly Active"[Mesh])
**#5**	#1 AND #2 AND #3 AND #4
Scopus	
	(HIV OR HIV/AIDS OR AIDS OR “HUMAN IMMUNODEFICIENCY” OR “ACQUIRED IMMUNODEFICIENCY)” AND TITLE-ABS-KEY (TASK-SHIFTING OR TASKSHIFTING OR (TASK* AND SHIFT*) OR TASK* OR (REFERR* AND (NURSE* OR PHARMAC*))) AND TITLE-ABS-KEY (ANTIRETROVIRAL OR ANTI-RETROVIRAL OR ART OR CART OR HAART) AND TITLE-ABS-KEY (RANDOM* OR RANDOMIZED OR RANDOMISED OR TRIAL OR COHORT* OR GROUP* OR COMPAR* OR OBSERVATIONAL OR PROSPECTIVE* OR RETROSPECTIVE* OR “SYSTEMATIC REVIEW” OR “META-ANALYSIS)”
Web of Science	
	(TS=(HIV OR HIV/AIDS OR AIDS OR “HUMAN IMMUNODEFICIENCY” OR “ACQUIRED IMMUNODEFICIENCY)” AND TS=(TASK-SHIFTING OR TASKSHIFTING OR (TASK* AND SHIFT*) OR TASK* OR (REFERR* AND (NURSE* OR PHARMAC*))) AND TS=(ANTIRETROVIRAL OR ANTI-RETROVIRAL OR ART OR CART OR HAART) AND TS=(RANDOM* OR RANDOMIZED OR RANDOMISED OR TRIAL OR COHORT* OR GROUP* OR COMPAR* OR OBSERVATIONAL OR PROSPECTIVE* OR RETROSPECTIVE* OR “SYSTEMATIC REVIEW” OR “META-ANALYSIS)”) OR (TI=(HIV OR HIV/AIDS OR AIDS OR “HUMAN IMMUNODEFICIENCY” OR “ACQUIRED IMMUNODEFICIENCY)” AND TI=(TASK-SHIFTING OR TASKSHIFTING OR (TASK* AND SHIFT*) OR TASK* OR (REFERR* AND (NURSE* OR PHARMAC*))) AND TI=(ANTIRETROVIRAL OR ANTI-RETROVIRAL OR ART OR cART OR HAART) AND TI=(RANDOM* OR RANDOMIZED OR RANDOMISED OR TRIAL OR COHORT* OR GROUP* OR COMPAR* OR OBSERVATIONAL OR PROSPECTIVE* OR RETROSPECTIVE* OR “SYSTEMATIC REVIEW” OR “META-ANALYSIS)”)
CENTRAL	
	HIV* OR HIV-1* OR HIV-2* OR HIV1 OR HIV2 OR HIV INFECT* OR HUMAN IMMUNODEFICIENCY VIRUS OR HUMAN IMMUNEDEFICIENCY VIRUS OR HUMAN IMMUNE-DEFICIENCY VIRUS OR HUMAN IMMUNO-DEFICIENCY VIRUS OR HUMAN IMMUN* DEFICIENCY VIRUS OR ACQUIRED IMMUNODEFICIENCY SYNDROME OR ACQUIRED IMMUNEDEFICIENCY SYNDROME OR ACQUIRED IMMUNO-DEFICIENCY SYNDROME OR ACQUIRED IMMUNE-DEFICIENCY SYNDROME OR ACQUIRED IMMUN* DEFICIENCY SYNDROME in Title, Abstract, Keywords and (TASK-SHIFTING OR TASKSHIFTING OR (TASK* AND SHIFT*) OR TASK* OR (REFERR* AND (NURSE* OR PHARMAC*))) in Title, Abstract, Keywords and ANTIRETROVIRAL OR ANTI-RETROVIRAL OR ART OR cART OR HAART in Title, Abstract, Keywords
WHO Global Health Library	
	(TASK-SHIFTING OR TASKSHIFTING OR (TASK* AND SHIFT*) OR TASK* OR (REFERR* AND (NURSE* OR PHARMAC*)) AND (HIV* OR human immunodeficiency) AND (antiretroviral OR anti-retroviral))) OR (HIV AND task-shifting) OR (HIV* AND task* AND shift*)

### Conference databases

We will search conference abstract archives on the web sites of the Conference on Retroviruses and Opportunistic Infections (CROI), the International AIDS Conference (IAC) and the International AIDS Society Conference on HIV Pathogenesis, Treatment and Prevention (IAS), for all available abstracts presented at these conferences from 1996 to the search date.

### Searching other resources

We will also search the references of relevant articles as well as the WHO International Clinical Trials Registry Platform (ICTRP) and Clinicaltrials.gov. We will contact relevant experts or organisations who may be aware of additional studies in this field.

### Data collection and analysis

We will base the methodology for data collection and analysis on the guidance provided in the Cochrane Handbook of Systematic Reviews of Interventions.^19^

### Selection of studies for inclusion

Two authors will read and assess the abstracts of identified publications for potentially eligible studies. We will obtain full text articles for all abstracts judged by at least one of the two authors, to be potentially eligible. Two authors will independently inspect these potentially eligible publications to establish the relevance of the article to the review according to the pre-specified criteria regarding study design, participants, interventions and outcome measures.

### Data extraction and management

Two authors will independently extract data into a pre-piloted data extraction form. The following characteristics will be extracted from each included study:

*Study details*: Complete citations of publications associated with the study, start and end dates, location, study design characteristics, type of facility involved, investigators, funding sources, recruitment, method of randomisation, sequence generation, method of allocation concealment, blinding of participants and personnel, blinding of outcome assessment, length of follow-up, losses to follow-up, withdrawals or drop-outs and other relevant details.

*Details of the intervention*: training of the cadre of health workers who were dispensing or distributing ART, what training or other support or supervision they received and other relevant details.

*Details of participants*: Trial inclusion and exclusion criteria, numbers of participants entering the trial, sex, clinical staging, CD4 count and other pertinent details.

*Outcome details*: Definitions of outcomes, details of how outcomes were assessed, numerators and denominators associated with each outcome, completeness of outcome data, effect estimates reported and other relevant outcome information.

### Assessment of risk of bias in included studies

We will assess the risk of bias in RCTs using the Cochrane risk of bias assessment tool for randomised studies.^19^ For non-RCT studies, we will use the Cochrane Risk of Bias Assessment Tool for Non-Randomised Studies of Interventions (ACROBAT-NRSI).^20^

We will resolve any disagreements between the authors conducting duplicate independent screening of search outputs, assessments of study eligibility, extraction of data, and risk of bias assessment by discussion and consensus. Should this fail to resolve the differences, a third author will arbitrate.

### Measures of effect

We will calculate and report risk ratios for dichotomous and time-to-event data and mean differences for continuous data with their 95% CIs.

### Unit of analysis issues

The unit of analysis will be the individual study participant. Cluster-randomised trials will be included in meta-analyses only after adjustments are made for design effect. Design effects for cluster-randomised studies will be corrected by using standard procedures, using the formula: design effect=1+(m−1)r, where m is the average cluster size and r is the intra-cluster correlation coefficient.

### Dealing with missing data

We will contact study authors if it is necessary to obtain data missing from published reports.

### Assessment of heterogeneity

We will examine statistical heterogeneity between study results using the χ^2^ test of homogeneity, with a signiﬁcance α-level of 0.1. In addition, we will use the I^2^ statistic to measure the amount of heterogeneity among the trials in each analysis. If we identify significant heterogeneity (ie, p<0.1), we will explore it by pre-specified subgroup analysis. If heterogeneity persists, we will perform sensitivity analyses, report results separately and propose reasons for the observed heterogeneity.

### Assessment of reporting biases

If any meta-analysis in our review includes 10 or more studies, we will assess the potential for publication bias using a funnel plot.^21^ We will attempt to minimise the potential for publication bias through a comprehensive search of published and unpublished literature.

### Data synthesis

We will conduct meta-analysis, if appropriate, using the Cochrane Review Manager software (RevMan [Computer program] The Nordic Cochrane Centre, *The Cochrane Collaboration, Copenhagen*, 2015; Version 5.3). If we find no significant statistical heterogeneity of effects, we will use the fixed effect method of meta-analysis. Otherwise, we will use the random effects model.

### Subgroup analysis

In pooled results with significant statistical heterogeneity, we will explore the cause of the heterogeneity through subgroup analyses, with subgroups defined by type of intervention (eg, cadre of health provider), comparison group and region of study (eg, sub-Saharan Africa, Southeast Asia, etc).

### Sensitivity analysis

We will conduct a sensitivity analysis to investigate the effect of excluding studies with high risk of bias, with a focus on bias introduced by inadequate allocation concealment, inadequate blinding of outcome assessment and substantial losses to follow-up.

### Certainty of evidence

We will assess the certainty (or quality) of evidence using the Grading of Recommendations, Assessment, Development and Evaluation (GRADE) approach,^22^ which defines the certainty of evidence for each outcome as “the extent of our confidence that the estimates of effect are correct”.^19^ The quality rating across studies has four levels: high, moderate, low and very low. Randomised trials are considered to be of high quality but can be downgraded for any of five reasons: risk of bias, indirectness of evidence, unexplained heterogeneity of effects, imprecision of effect estimates and high probability of publication bias. Similarly, observational studies are considered to be of low quality, but can be upgraded for any of three reasons. The quality level of a body of evidence can be increased if there is a large magnitude of effect, if all plausible confounding would reduce a demonstrated effect, and if there is a dose-response gradient.

### Reporting of this review

The findings of this review will be presented in a number of ways. The study selection process will be summarised using a flow diagram, and if we identify 10 or more eligible studies, we will assess publication bias using funnel plots. Where appropriate, we will use risk of bias graphs, forest plots and GRADE summary of findings tables. The non-quantitative outcomes will be reported descriptively. We will provide tables of both included and excluded studies. We have prepared this protocol as recommended by the PRISMA-P (Preferred Reporting Items for Systematic Review and Meta-Analysis Protocols) guidelines^23^ and will report the findings of the review as recommended by the PRISMA statement.^24^

## Ethics and dissemination

Since systematic reviews do not directly involve human participants, they do not require ethical clearance.^25^ We will provide the findings of this review to the WHO, with the hope that they may guide policy recommendations of this normative agent regarding the shifting of ART dispensing or distribution from pharmacy to non-pharmacy personnel. Although the majority of national programmes in low and middle-income countries have adopted task shifting in ART care at different levels, there has been no global policy to guide the practice for task shifting from pharmacy to non-pharmacy personnel. We will also publish the findings of the systematic review in a peer-reviewed journal.
